# Nanocomposite hydrogel actuators hybridized with various dimensional nanomaterials for stimuli responsiveness enhancement

**DOI:** 10.1186/s40580-019-0188-z

**Published:** 2019-06-10

**Authors:** Im Kyung Han, Taehun Chung, Jihoon Han, Youn Soo Kim

**Affiliations:** Department of Materials Science and Engineering, Pohang University of Science and Technology (POSTECH), 77 Cheongam-Ro, Nam-Gu, Pohang, Gyeongbuk 37673 Republic of Korea

**Keywords:** Hydrogels, Actuators, Nanocomposite materials, Stimuli-responsive, Anisotropy, Soft materials

## Abstract

Hydrogel actuators, that convert external energy, such as pH, light, heat, magnetic field, and ion strength, into mechanical motion, have been utilized in sensors, artificial muscles, and soft robotics. For a practicality of the hydrogel actuators in a wide range of fields, an establishment of robust mechanical properties and rapid response are required. Several solutions have been proposed, for example, setting porous and anisotropy structures to hydrogels with nanocomposite materials to improve the response speed and deformation efficiency. In this review paper, we focused on hydrogel actuators including various nanocomposite by categorizing the dimensional aspects of additive materials. Moreover, we described the role of diverse additive materials in terms of the improvement of mechanical property and deformation efficiency of the hydrogel actuators. We assumed that this review will provide a beneficial guidance for strategies of developing nanocomposite hydrogel actuators and outlooks for the future research directions.

## Background

Hydrogels, which are a three-dimensional (3D) network of cross-linked hydrophilic polymer chains with high water content (up to 90 wt%), are highly elastic and soft materials. If these hydrogels contain stimuli-responsive polymer, they can produce drastic changes in their volume in response to environmental stimuli, such as heat, light, and magnetic and electric fields. Particularly, hydrogel actuators, converting the energy received from outside into mechanical motion, can exhibit soft and flexible motions similar to that of living creatures. Additionally, actuators with rigid materials (e.g., metals) require joints to connect the rigid parts together, whereas hydrogel actuators do not require these joints. Owing to the flexibility, biocompatibility, and stimuli sensitivity advantages of hydrogels, they can be utilized in a wide variety of applications, including drug delivery [1–6], smart window [7, 8], and soft actuators [8–15].

There are various types of external stimuli including pH [16, 17], light [18, 19], heat [20], magnet field [21, 22], and ion strength [23, 24]. Stimuli-responsive polymers containing hydrogels change their hydrophilicity in response to these external stimuli to ensure that hydrogels demonstrate macroscopic shrinking or swelling by expelling or absorbing water molecules. However, typical hydrogels exhibit a sluggish mechanical change in response to these external stimuli because their volume phase transitions are associated with diffusion and mass transport of solvent molecules in both the interior and exterior of the hydrogel network. Generally, stimuli are externally applied to inside, and the surface of the hydrogel forms skin layers that interfere with the permeation of solvent molecules. These skin layers are also one of the main factors causing the delay in shrinking or swelling of the hydrogel [25]. Reducing the size of the gel or introducing a porous structure in the hydrogel network could accelerate the response rate [26–28].

Synthetic hydrogels typically comprise randomly oriented 3D polymer networks, physically or chemically cross-linked polymers. Meanwhile, biological systems employ anisotropic structures in hierarchically integrated building units. These anisotropic structures often play a crucial role in biological systems for performing a specific function, as represented by muscle tissue containing unidirectionally oriented actin–myosin units. If a synthetic polymer system can be used to achieve these well-oriented structures, developing highly efficient directional action of the hydrogel, which is similar to muscle contraction, and new biomimetic materials would be possible.

**Fig. 1:**
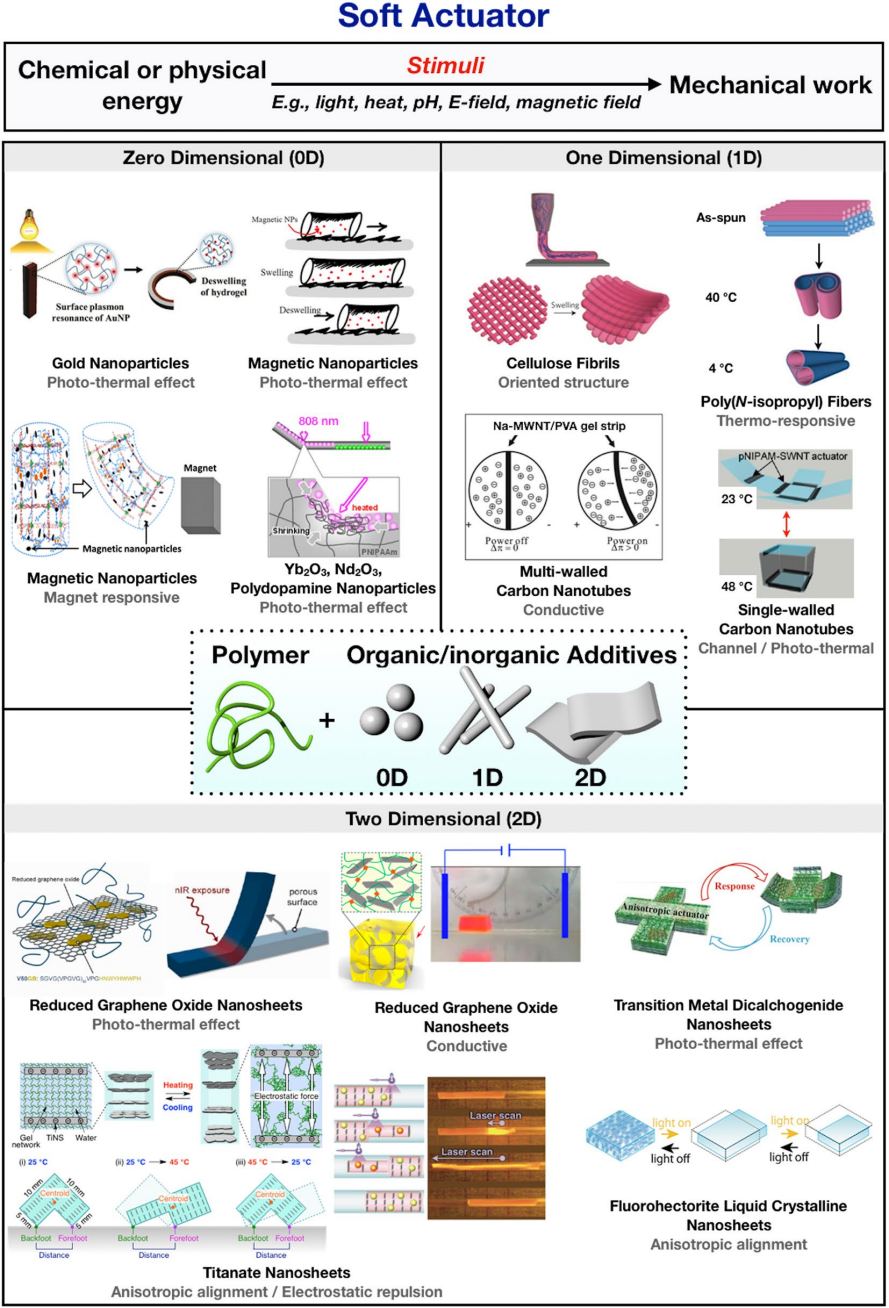
Nanocomposite hydrogel actuators classified with dimensions of additive organic/inorganic materials

To satisfy rapid response, high efficiency, and directional motion of the hydrogel actuation, the most extensively used approach is embedding zero-dimensional (0D)-, one-dimensional (1D)-, or two-dimensional (2D)-shaped additives in the hydrogel. By utilizing functional nanomaterials as additives, the performance of the hydrogel actuators could be dramatically improved and/or diversified. Figure 1 shows the summary of the overall categorization of the nanocomposite hydrogel actuators. We separated the additives in 0D-, 1D-, and 2D nanomaterials.

Various metal nanoparticles have been utilized as useful 0D additives. For example, gold nanoparticles (AuNPs) and iron oxide nanoparticles (IONPs) are generally known to generate thermal energy owing to the surface plasmon resonance (SPR) and magneto-thermal effects, respectively. Additionally, rare-earth oxide nanoparticles (REO NPs), such as ytterbium oxide, neodymium oxide, and poly-dopamine nanoparticle (PDA-NPs), have been embedded in the hydrogels to exploit their particular functions. Nanofibers and carbon nanotubes (CNTs) are representative materials of 1D additives. The aligned nanofibers assist the hydrogel actuator to deform directionally. CNTs can also generate thermal energy by absorbing near-infrared (NIR) light and possess high electric and thermal conductivity. In the case of 2D additives, photo-thermal reactive and electro-conductive graphene oxides (GOs) and transition metal dichalcogenides (TMDs) have been utilized extensively. Titanate nanosheets (TiNSs) are implanted in the hydrogels for the electrostatic repulsion between TiNSs. Furthermore, other types of nanosheets, such as fluorohectorite liquid crystal nanosheets (FHT LC NSs) and alumina platelets, have been employed as anisotropic reinforcements for the directional deformation.

In this review, the selected examples of the recently reported nanocomposite hydrogel actuators are classified into the types of additives used. Moreover, the roles of each additive in the enhanced performance of the hydrogel actuators are discussed (Fig. 1). We expect that this review paper provides rational strategies for the development of artificial muscles by using functional nanomaterials and the hydrogels.

## Review

### 0D nanocomposite hydrogel actuators

0D nanomaterials, with all their dimensions measured within the nanoscales, are commonly referred to as spherical nanoparticles or nanoclusters. The 0D nanomaterials of polymers, metals [29, 30], metal oxides [31, 32], and semiconductor materials [33] have particular properties (e.g., magnetic, optical, and electronic) owing to their nanoscale dimensions. By using these properties, 0D nanomaterials can be utilized as energy converters, resulting in the actuation of hydrogels. In this section, the hydrogel actuators are classified according to their embedded nanoparticle elements, such as gold [34–36], iron oxide [21, 22, 37, 38], and REO nanoparticles [39, 40].

#### AuNPs

Noble metal nanoparticles are generally known to possess strong high-energy absorption due to inherent interband transitions and to absorb SPR light. Therefore, AuNPs can convert light energy into thermal energy.

**Fig. 2:**
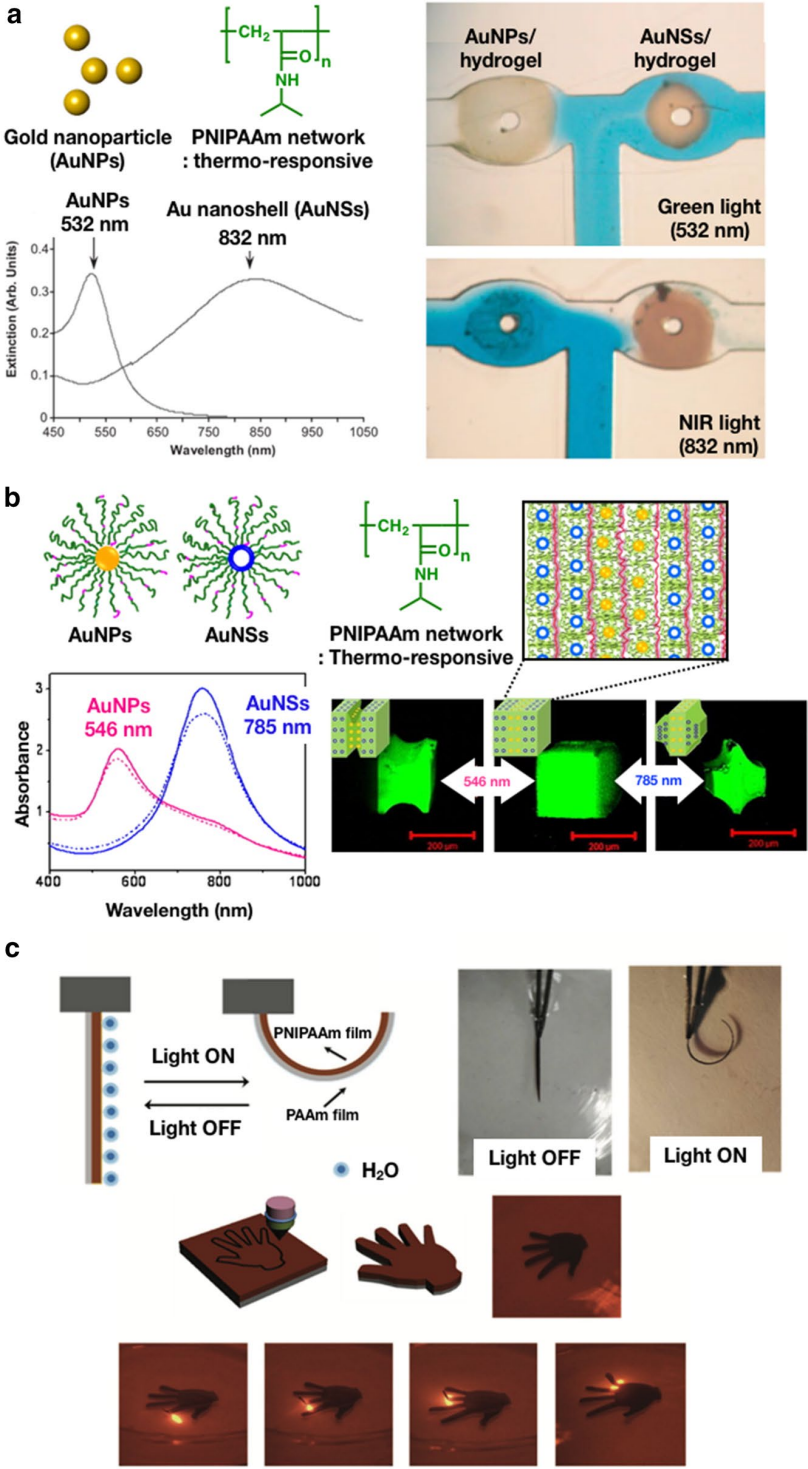
**a** Excitation wavelength of AuNPs and AuNSs (left) and the T-junction demonstration of the microfluidic nanovalve device with PNIPAAm/AuNPs and AuNSs hydrogels comprising 100-µM-wide channels. (Reproduced with permission from Ref. [35], © 2005, WILEY–VCH Verlag GmbH & Co. KGaA, Weinheim). **b** Schematic of the AuNPs and AuNSs modified with PNIPAAm, and confocal laser scanning microscopy (CLSM) images of light-controlled shape changes in three-strata AuNSs_300_–AuNPs_300_–AuNSs_300_ cubes upon irradiation using wavelengths at 546 nm (1.1 W/cm^2^) and 785 nm (2 W/cm^2^). (Reproduced with permission from Ref. [42], © 2012 American Chemical Society). **c** Schematic of the volume shrinking of the hydrogels with AuNPs under light irradiation (top), actuation mechanism responding to visible light, and optical photos of the PNIPAAm-AuNPs/PAAm actuator without light irradiation (left panel) and with light irradiation (150 mW/cm^2^, right panel) for 25 s. Images of the photo-thermal hydrogel actuator performing a precise finger-like one-by-one bending via light (bottom). (Reproduced with permission from Ref. [36], © 2017, WILEY–VCH Verlag GmbH & Co. KGaA, Weinheim)

With this knowledge, Sershen et al. [35] reported an optically controllable hydrogel valve in a microfluidic device, where AuNPs were immobilized in thermosensitive polymers, such as poly(*N*-isopropylacrylamide) (PNIPAAm) (Fig. 2a). PNIPAAm is the most investigated thermosensitive polymer in hydrogel actuators that exhibits sharp, reversible phase transitions in water at approximately 32 °C [41]. The composite hydrogels were prepared by first mixing the AuNPs with a monomer solution and then fixing AuNPs within the hydrogel matrix via cross-linking radical polymerization. For a differential control of the hydrogel actuation in response to light wavelengths, AuNPs and gold nanoshells (AuNSs) with distinct and strong optical absorption profiles were used. By irradiating appropriate light wavelengths, the temperature within the hydrogel could rise above the lower critical solution temperature (LCST) of PNIPAAm, resulting from the photo-thermal effects of AuNPs. Based on the characteristics of the AuNPs composite hydrogels, a remote-controllable valve of a microfluidic device at a T-junction was developed. At 532 nm irradiation, AuNPs with a plasmon peak at 532 nm lead to the collapse of the PNIPAAm network, resulting in the shrinkage of the hydrogel and, thus, opening of the valve. However, this irradiation did not result in the collapse of the hydrogel containing AuNSs. Thus, the valve comprising the composite hydrogel with AuNSs remained closed, as shown in Fig. 2a. The composite hydrogels responded in less than 5 s. Furthermore, the response time can be reduced by increasing the light intensity.

Sukhishvili et al. [42] demonstrated a layered nanocomposite undergoing a spatially anisotropic deformation by the light irradiation. AuNPs and AuNSs were grafted with PNIPAAm brushes and assembled using a layer-by-layer technique, as shown in Fig. 2b. By combining AuNPs and AuNSs in various layers, the shrinkage of certain regions of the hydrogel can be controlled at specific excitation wavelengths. Figure 2b also shows the microcubes of the layered composite hydrogel exhibiting partial shrinkage at light irradiation of 1.1 W/cm^2^ 546 nm or 2 W/cm^2^ 785 nm. At 546 nm irradiation, the shrinking of the center layer could be selectively achieved, whereas the outer layer collapsed at 785 nm irradiation. The shape change of the composite hydrogel reached the equilibrium after 15 min and was restored to its original state at approximately 5 s after the laser was turned off and was also highly reversible.

Shi et al. [36] also designed a photo-thermal hydrogel actuator that performed a precise finger-like one-by-one bending via light irradiation. To achieve the bending motion during light irradiation, hydrogel bilayers, comprising nanocomposite PNIPAAm hydrogel with AuNPs and non-thermoresponsive poly(acrylamide) (PAAm), were prepared by using a layer-by-layer strategy. The bending motion occurred due to the mismatch of expansion coefficients between the PNIPAAm and PAAm layers. Figure 2c illustrates that the resulting nanocomposite actuators exhibited flexible, reversible bending and non-bending movements owing to local light irradiation. When the actuator was irradiated with light at a wavelength of 411 nm, it showed a maximum curvature of 4.28 cm^−1^ in 24 s.

Based on these results, the PNIPAAm hydrogels with AuNPs can reveal the photo-induced actuations, wherein AuNPs rapidly and effectively converted the light energy absorbed by SPR into heat energy. Hence, these AuNP-based hydrogel actuators have been proposed to be ideal candidates for various applications, such as in light-driven soft robots and artificial muscles.

#### IONPs

Generally, magnetic nanoparticles (e.g., IONPs) have been used in various fields, such as MRI reagent [43] and hyperthermia [44]. Owing to the magnetic moment of the IONPs, they could be aligned by applying magnetic field and exhibit a magneto-thermal effect through the alternating magnetic field (AMF). When these unique characteristics are applied to the hydrogel actuators, remote-controllable actuation of the hydrogels by using a magnet could be obtained. This section discusses various studies on hydrogel actuators that are combined with IONPs.

**Fig. 3:**
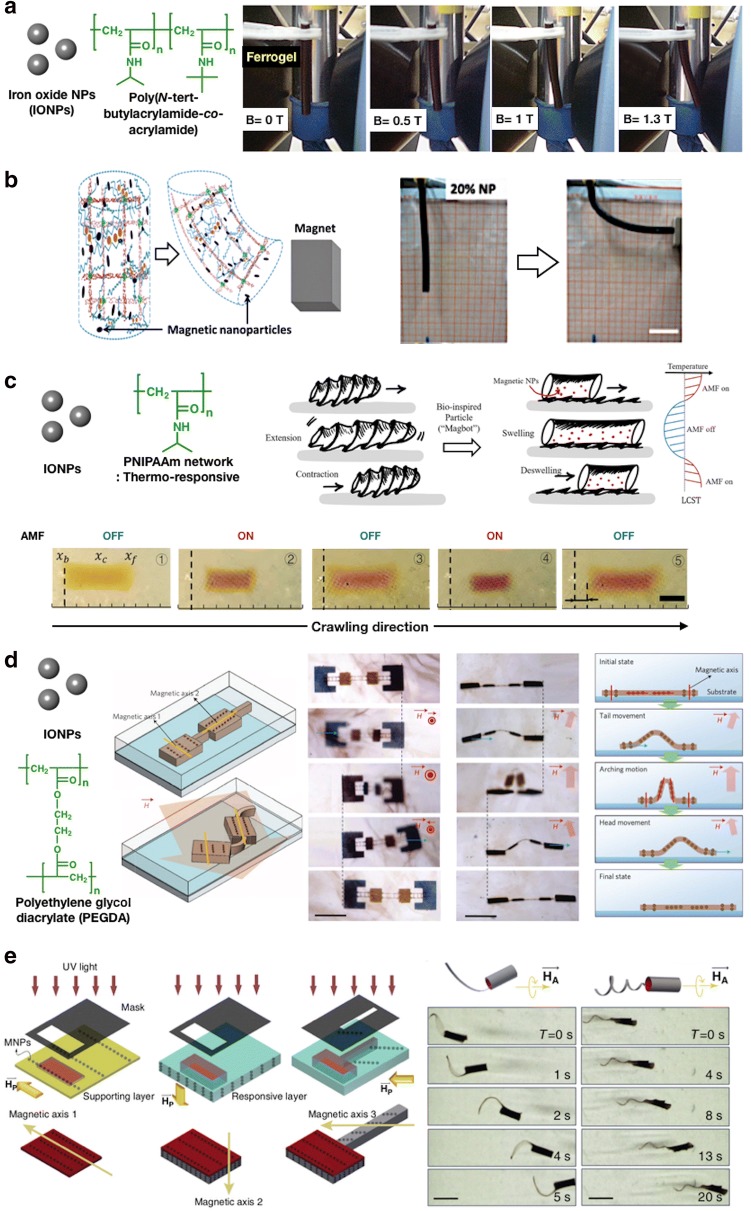
**a** Images of the bending process in the magnetic field of the ferrogel (Reproduced with permission from Ref. [45], © 2009, Inc. J Appl Polym Sci, Wiley Periodicals). **b** A cantilever actuator made of magnetic hydrogel (left) and its demonstration of the magnetic responses with 20% IONPs content (right). (Reproduced with permission from Ref. [37], © 2015, The Royal Society of Chemistry). **c** Schematic of the crawling motion of the hydrogel actuator under the AMF, similar to that of maggots. (Reproduced with permission from Ref. [46], © 2017, The Authors; published by Springer Nature). **d** Demonstration of the magnetic actuator showing sequential movement of the microlooper. The movement of the magnetic actuator possesses two different magnetic axes. Therefore, it actuates in a zigzag conformation when the magnetic field is applied. It is synthesized with PEGDA hydrogel. (Reproduced with permission from Ref. [38], © 2011, Macmillan Publishers Limited). **e** Schematic of the synthesizing method of the biomimetic soft micromachines with photo-patterning fabrication (left). The image also shows the optical image with different types of complex micromachines driven by the rotating uniform magnetic fields. (Reproduced with permission from Ref. [47], © 2016, Nature Publishing Group)

Caykara et al. [45] reported a hydrogel actuator referred to as “ferrogel.” A two-step process was required to synthesize the ferrogel. First, the hydrogels of poly(*N*-*tert*-butylacrylamide-*co*-alkylamide) [P(NTBA-*co*-AAm)] were synthesized. Second, magnetite (Fe_3_O_4_) particles were formed by *co*-precipitation of Fe(II) and Fe(III) ions in an alkaline medium at 70 °C. The result showed that the degree of bending of the ferrogel is dependent on the strength of the applied magnetic field. Moreover, the transition between straight and curved forms of the ferrogel occurred simultaneously with on/off switching of the magnetic field (Fig. 3a).

However, most conventional magnetic hydrogels are weak and fragile. To address this limitation, Haider and Yang [37] developed highly stretchable and exceptionally tough magnetic hydrogels with physically and chemically cross-linked network between the dispersed alginate-coated Fe_3_O_4_ nanoparticles and PAAm (Fig. 3b). A permanent NdFeB magnet was used to activate the cylindrical hydrogel containing 20 wt% of Fe_3_O_4_. Specifically, the higher the nanoparticle content, the better the magnetic properties of the hydrogel. However, the mechanical properties of the hydrogel, such as toughness and stretchability, become worse. Thus, it remains a remarkable challenge to balance the mechanical and magnetic properties of the magnetic hydrogel.

Shen et al. [46] developed a soft hydrogel crawler that exhibited a directional movement in an enclosed space, similar to maggot movement. The hydrogel consisted of PNIPAAm and dispersed IONPs, and its periodic movement was demonstrated by heating the embedded IONPs through Brownian and Neel relaxation using AMF. Thus, when the AMF was turned on, the hydrogel was heated above the LCST and then collapsed. When the AMF is turned off, the hydrogel cooled down below the LCST and then restored to its original volume. If the gel was placed in a confined chamber with an asymmetric surface (e.g., ratchet), the friction coefficient in forward and backward directions varied. This function preferentially slid the hydrogel in the direction of the lowest friction (Fig. 3c).

An actuator using a non-uniform magnetization profile (Fig. 3d) was first introduced by Kim et al. [38]. The researchers constructed the magnetically programmable polymer composite actuators by confining self-assembled IONPs in a polymer matrix. Here, the aim was to demonstrate the spatially modulated photo-patterning of the self-assembled IONPs with stronger magnetization of paramagnetic materials. By repeatedly tuning and confining the assembly of IONPs through photopolymerization, the microactuator was manufactured wherein all its portions move in different directions at a uniform magnetic field. They demonstrated a polymer nanocomposite actuator capable of 2D and 3D complex actuations (e.g., caterpillar movement) in which conventional microactuators could not achieve. By selecting the appropriate magnetic field direction and strength for the desired configuration, the actuator could obtain the accurate movement of a microlooper.

Huang, Sakar, and colleagues [47] developed a rapid prototyping process inspired by origami to construct a self-powered micromachine with complex body planning, reconfigurable shape, and controllable mobility (Fig. 3e). The research was focused on bio-inspired corkscrew movement and was not limited to the propulsive force generated by the rotating passive flagellum. Highly complex swimming strategy could be realized by engineering with different magnetic axes and applying a time-varying magnetic field. The operation of a flat flagellum micromachine remarkably differs from that of a helical flagellum micromachine. Although the helical flagellum generated propulsion force by breaking the time reversal symmetry, the planar flagellum acted similar to a flexible oar that transformed the entire body, thereby causing the forward movement.

#### Other nanoparticles

**Fig. 4:**
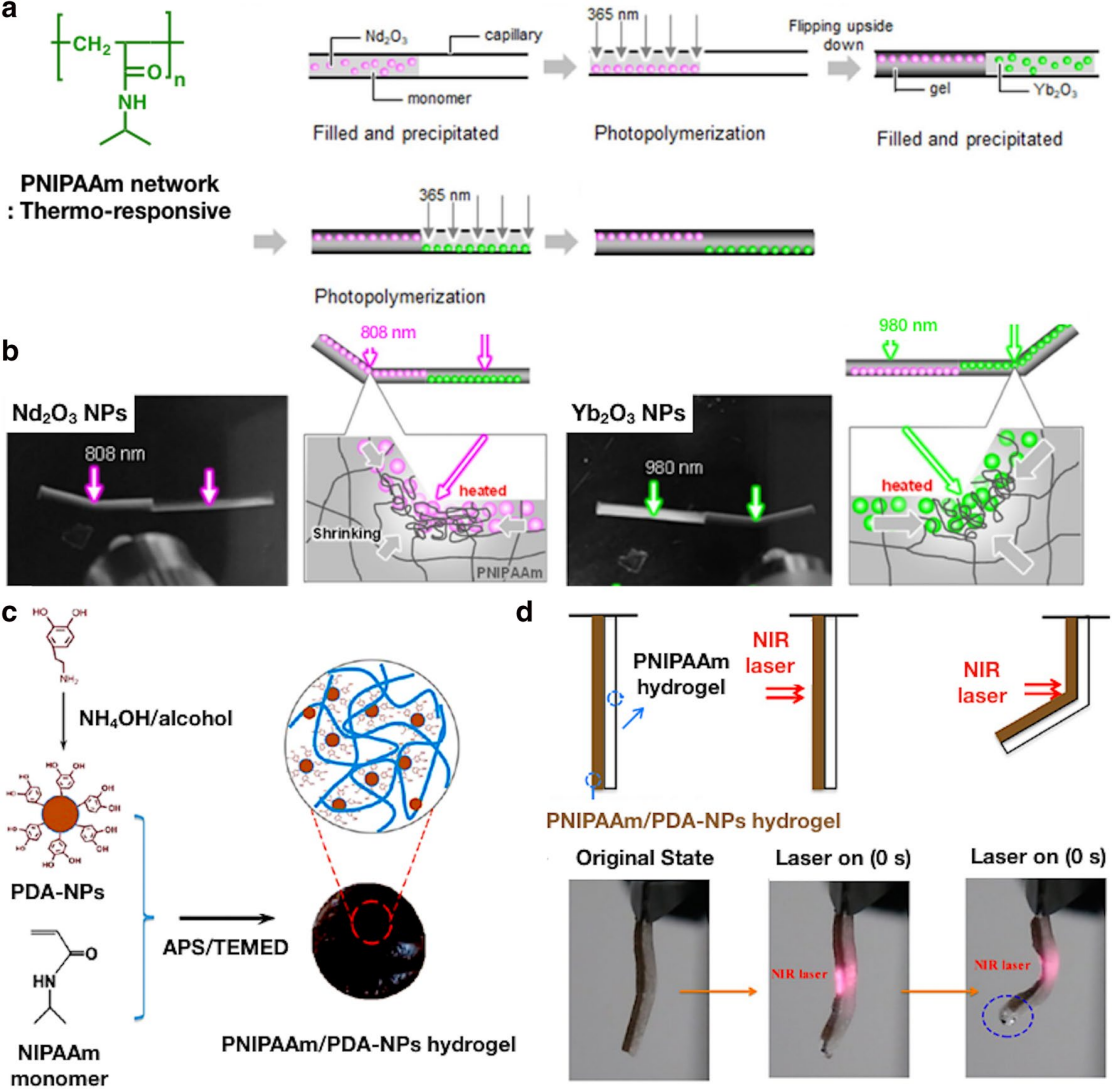
**a** Schematic of the synthesis of REO NPs combined with rod shaped hydrogels for bending actuation. **b** Images of the as-prepared rod shaped hydrogels with 808 nm (Nd_2_O_3_ NPs selective absorbance) and 980 nm (Yb_2_O_3_ NPs selective absorbance) irradiation demonstrating the bending actuation. This NIR light irradiation exploited at 3 W/cm^2^ for 60 s at 28 °C. (**a**, **b** were reproduced with permission from Ref. [39], © 2018, Inc. The Authors; published by Springer Nature). **c** Schematic of the synthesis of PNIPAAm/PDA-NPs hydrogel by the oxidative self-polymerization of PDA-NPs and free radical polymerization of PNIPAAm. **d** Bending motion of the bilayered hydrogel actuator, composed of PNIPAAm/PDA-NPs and PNIPAAm hydrogel layers, upon NIR laser irradiation. (**c**, **d** were reproduced with permission from Ref. [40] © 2016, American Chemical Society)

REO NPs are often used as fluorescent, phosphorescent, and upconverting photoluminescent materials. Additionally, the excited energy must be emitted through thermal radiation to be used in light-to-heat conversion applications. Watanabe et al. [39] reported photo-responsive actuators that utilize two different IR responsive particles. In their research, PNIPAAm hydrogels were synthesized with REO NPs, such as neodymium(III) oxide (Nd_2_O_3_) and ytterbium (III) oxide (Yb_2_O_3_) particles (Fig. 4a). Given that Nd_2_O_3_ and Yb_2_O_3_ particles possess independent narrow IR absorption at 808 and 980 nm, respectively, the surroundings of each particle were individually heated through irradiation at each adsorption wavelength, leading to local volume phase transition of the PNIPAAm hydrogel (Fig. 4b). The researchers demonstrated that a rod shaped hydrogel reached a constant bend within 10 s under IR irradiation. After the light was turned off, it gradually restored to its original shape within a few minutes.

Han et al. [40] incorporated PDA-NPs, possessing NIR-responsive ability, to the PNIPAAm hydrogel to develop the photo-thermal responsive actuators (Fig. 4c). They synthesized a bilayered hydrogel with PNIPAAm/PDA-NPs layer and a pure PNIPAAm layer to demonstrate the actuation under NIR irradiation (808 nm). After the 30 s irradiation of NIR light, the PNIPAAm/PDA-NPs layer distinctively collapsed, causing bending motion of the bilayered hydrogel (Fig. 4d) [48].

### 1D nanocomposite hydrogel actuators

1D nanomaterials refer to a material that has only one dimension at a nanometric scale and their two other dimensions are larger than the nanometric scale. Unlike bulk materials, their physical and chemical properties have elicited attention academically and practically. Presently, various manufacturing methods for synthesizing diverse structures of these 1D nanomaterials have been proposed. This section will describe the nanocomposite hydrogel actuators containing nanofibers [49–51] and CNTs [52, 53].

#### Nanofibers

Nanofibers can be defined as 1D flexible solid nanomaterials with a diameter of 100 nm and aspect ratio of 100:1 or higher. With the recent rapid development of nanomaterial-related technologies, thin nanofibers have been developed. Hence, many attempts of aligning the nanofibers have been executed to achieve sophisticated structures and anisotropic motions of the hydrogels. Interestingly, this phenomenon could be found in nature. For example, many plants are known to possess operational performance based on the local expansion behavior, resulting from directionally oriented cellulosic fibers. Inspired by this finding, hydrogel actuators that use fibrillated nanofillers have been investigated. By applying a shear force to the dispersion that contains nanofibers and monomers, the nanofibers could align in parallel direction to the shear direction. Furthermore, the anisotropic hydrogel can be obtained via in situ polymerization.

**Fig. 5:**
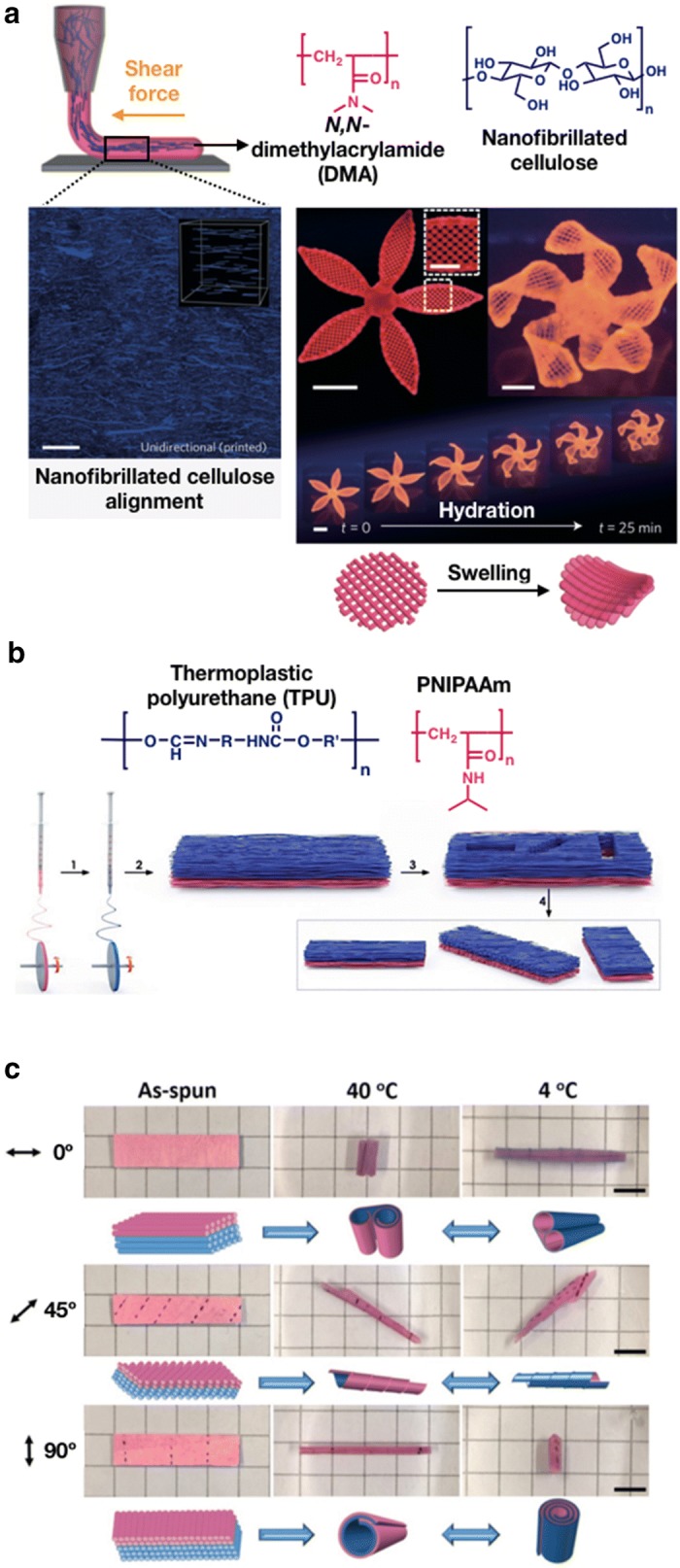
**a** Schematic of the synthesis of nanofibrillated cellulose aligned hydrogel via the shear-induced biomimetic 4D printing method. Photographs of the actuation of complex flower morphologies oriented with long axis of each petal, and with time lapse during the swelling process. (a was reproduced with permission from Ref. [50], © 2016 Macmillan Publishers Limited). **b** Schematic of the synthesis of bilayer actuators using a sequential electrospinning method. The actuator comprises TPU (blue) and PNIPAAm (pink) using a UV cross-linking method. The materials were cut in different angles to obtain various orientations. **c** The actuation of the bilayer hydrogel depends on diverse orientations of the fibers in water at different temperatures. (**b**, **c** were reproduced with permission from Ref. [51], © 2015, WILEY–VCH Verlag GmbH & Co. KGaA, Weinheim)

Gladman and Matsumoto [50] used viscoelastic solution that contains AAm monomer, photoinitiator, and cellulose nanofibers as ink for a printing system. During the printing process, these fibrils undergo shear-induced alignment as the ink flows through the nozzles, thereby producing printed filaments with anisotropic stiffness (Fig. 5a). After printing at ambient conditions, the acrylamide monomers were photopolymerized to ensure the generation of longitudinally expandable hydrogels. By using this technology, the researchers also printed a variety of plant-inspired architectures (Fig. 5b). This biomimetic four-dimensional (4D) printing offers an easy route to encoding complex shape changes in hydrogel-based composites materials.

Through electrospinning techniques, nanofiber-, nanorod-, and nanotube-shaped materials can be easily and cheaply produced. Liu et al. [51] addressed a fibrous bilayer system using thermoplastic polyurethane (TPU) and cross-linked PNIPAAm fibers. The TPU and PNIPAAm fibers were oriented at various angles as passive and active layers, respectively. Then, these fibers can display a pre-programmed rolling movement (Fig. 5c) with temperature change. It was demonstrated that reversible coiling, rolling, bending, and twisting motions in distinct directions for many cycles (at least 50).

#### CNTs

CNTs are cylinders of hexagons comprising six carbon atoms connected in a tubular shape. The thermal conductivity of CNTs is the same as that of diamonds, and the tensile strength exceeds that of diamonds. When these characteristics are applied to the hydrogel actuators, it is expected to improve of their current actuation performance or initiate new functions.

**Fig. 6:**
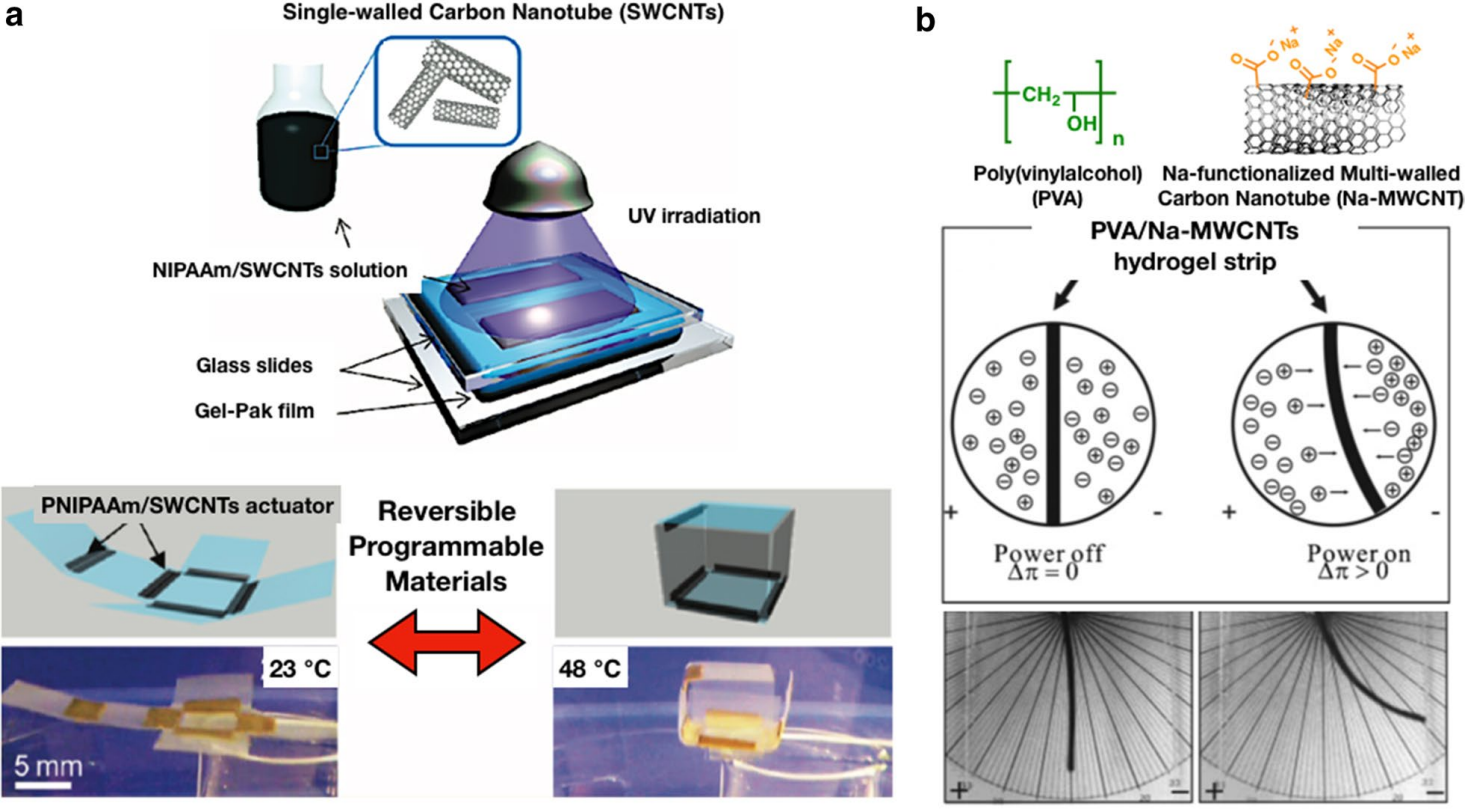
**a** Schematic of the PNIPAAm/SWCNTs hydrogel fabrication using Gel-Pak confinement channels and UV curing method. Reversible and programmable folding/unfolding of cube in response to temperature changes, resulting from the PNIPAAm/SWCNTs. (Reproduced with permission from Ref. [52], © 2011, American Chemical Society). **b** Images of the bent PVA/Na-MWCNTs hydrogel strip with 10%(w/w) containing Na-MWCNTs in 3 mM of Na_2_CO_3_ aqueous solution at 10 V/cm DC electric field. (Reproduced with permission from Ref. [53], © 2005, American Chemical Society)

Zhang et al. [52] have successfully constructed a 3D shape from a 2D PNIPAAm sheet using the origami approach. They demonstrated single-walled carbon nanotubes (SWCNTs) composite hydrogels (PNIPAAm/SWCNTs) in their study that exhibit up to five times thermal response time improvement when compared with conventional PNIPAAm hydrogels. The reason is SWCNTs generate a large number of porous structures in PNIPAAm hydrogels, resulting in water diffusion improvement. Additionally, SWCNTs are regarded as efficient channels for water flow driven by the osmotic pressure. Hence, only ∼ 2.7 s is required for the hydrogel hinge to reach a 90° angle, when compared with ∼ 14 s for the hydrogel without SWCNTs (Fig. 6a). Therefore, the PNIPAAm/SWCNTs programmable actuators are assumed to be employed in a number of new applications, such as smart solar tracking system or even tissue connectors for biological media.

For the multi-walled carbon nanotubes (MWCNTs)-based actuator, Shi et al. [53] first reported a new type of actuator based on PVA/Na-MWCNTs hydrogels (Fig. 6b). They suggested sodium functionalized to MWCNT-COO^−^ as polyelectrolytes additives. Generally, polymer electrolyte gels can reveal a partially swollen and shrunken region under the electric field, resulting in bending motion. In the PVA/Na-MWCNTs composite hydrogels, MWCNT-COO^−^ was regarded as a negatively charged polyanion. When the hydrogel strip was subjected to direct current (DC) electric field, sodium ions moved toward the cathode, but the polyanion MWCNT-COO^−^ did not move. Accordingly, the expansion of the hydrogel on the anode side was accompanied with that of contraction on the cathode side, assisted by the high electric conductivity of the MWCNT. These electroactive hydrogel actuators could be used in various applications, such as microswitches, artificial muscles, robotics, optical displays, and micro-pumps.

### 2D nanocomposite hydrogel actuators

2D nanosheets with nanosized thickness and infinite length on a plane emerge as novel materials owing to their special properties [54, 55]. Apart from graphene being a single layer of carbon atoms arranged in a 2D honeycomb pattern, other inorganic analogs, such as TMDs, metal oxides, and 2D compound sheets, have received a considerable attention. Particularly, oxide nanosheets have abundant structural diversity and electronic properties. Thus, they can be utilized in applications ranging from catalysis to electronics. One of the most important and attractive aspects of exfoliated nanosheets is their ability to develop a variety of nanostructures with 2D structural blocks. Currently, studies applying 2D materials to hydrogel actuators, such as GOs [56–59], TiNSs [60], TMD NSs [61, 62], FHT CL NSs [63, 64], and alumina platelets [65], are conducted actively. In this section, we will discuss distinctive features and roles of these 2D materials being integrated with hydrogel actuators.

#### GOs

GOs are one of the most extensively used 2D nanomaterials for fabrication of hydrogel actuators owing to their excellent mechanical, electrical, and optical properties [56–59]. Reduced GO nanosheets (rGOs) are obtained from the oxidation, exfoliation, and reduction of graphite. rGOs absorb NIR light and efficiently generates heat. Their photo-thermal efficiency is better than the unreduced GOs. The challenge to consider when using rGOs in an aqueous environment is its aggregation vulnerability due to its hydrophobicity. Therefore, functionalization of the rGOs surface is required.

**Fig. 7:**
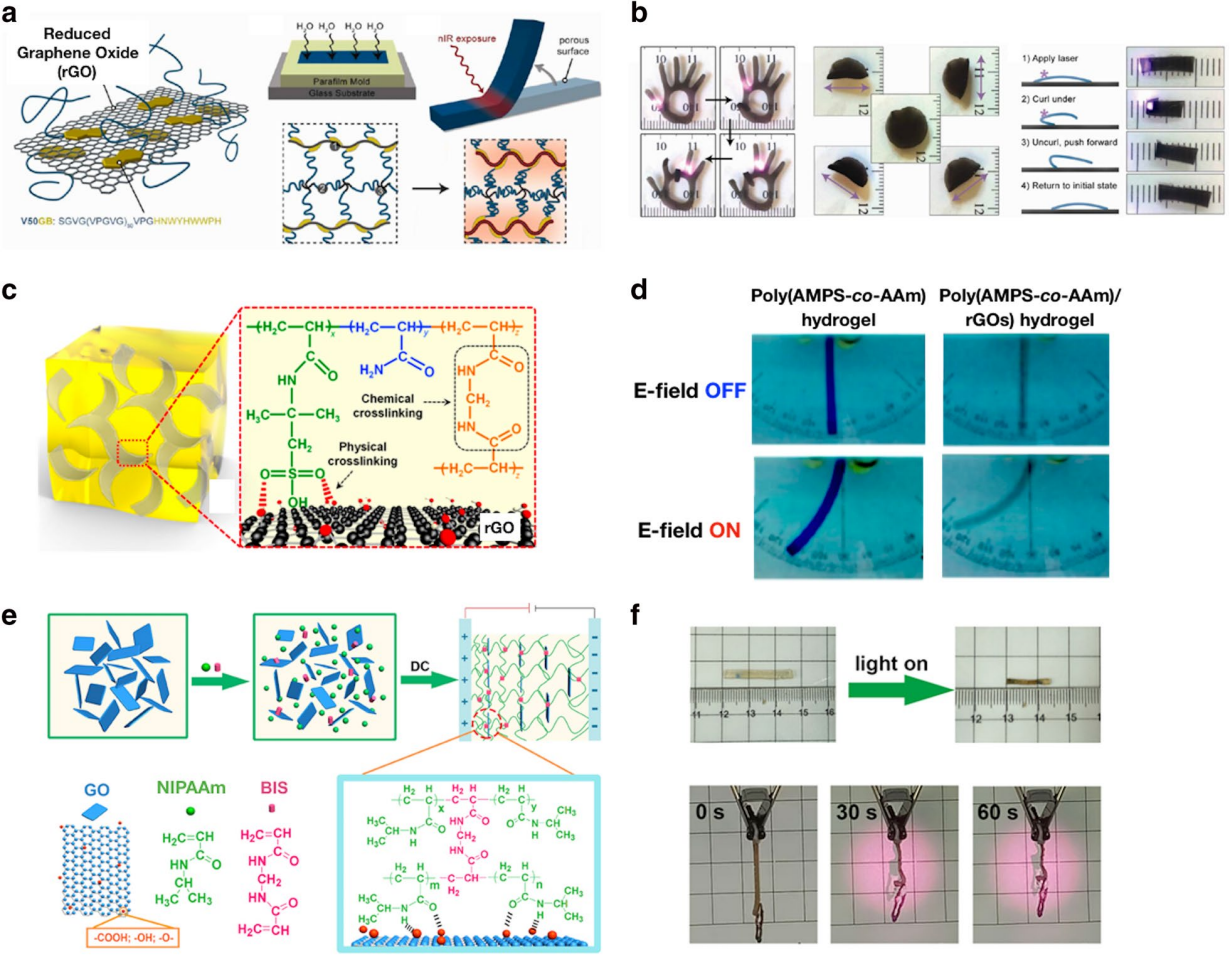
**a** Scheme of the synthesis of the V50 GB functionalized rGO based hydrogel. **b** Various actuations of the rGO hydrogel actuator via NIR light exposure such as light-driven bending and unbending of fingers, raster direction-dependent folding, and crawling. (**a**, **b** were reproduced with permission from Ref. [57], © 2013, American Chemical Society). **c** Schematic of the poly(AMPS-co-AAm)/rGO composite hydrogels. **d** Images of the poly(AMPS-co-AAm)/rGO hydrogel actuation with electric field of 10 V. (**c**, **d** were reproduced with permission from Ref. [58], © 2017, American Chemical Society). **e** Schematic images of the synthetic process to prepare the hydrogel containing gradually oriented GOs via the DC electric field. **f** Photographs of the NIR light-responsive hydrogel actuator before- and after irradiation for 50 s (top). The images demonstrate the lifting cargo of the hydrogel after NIR irradiation for 60 s (bottom). (e and f were reproduced with permission from Ref. [59], © 2018, American Chemical Society)

Wang et al. [57] reported NIR light-driven actuators by accommodating genetically engineered elastin-like polypeptides (ELPs) with rGOs. They chose the ELPs as a functional polymer because these polypeptides have controllable temperature responsiveness in aqueous solutions [66]. ELP is soluble in aqueous solution at below the transition temperature (Tt), and insoluble at above the Tt. Moreover, ELP is elastomeric proteins, so that cross-linked ELP may result in large and elastic volume change in response to temperature change with minimal energy loss [67]. Furthermore, ELP exhibits excellent biocompatibility [68]. The following procedure is used to fabricate the ELP–rGOs complex hydrogel. First, hybrid nanoparticles were prepared by functionalizing rGOs with a rationally designed ELP. Second, anisotropic microstructures were produced by cross-linking between hybrid nanoparticles in water. Third, the hydrogel was locally irradiated with NIR light to partially shrink the ELP network and induce a bending motion (Fig. 7a). As the laser intensity and rGOs concentrations increased, the bending speed and angle also increased. The bent hydrogels recovered up to 74–84% within 10 s after removing the NIR light. The hydrogel actuators that perform various mechanical operations have been developed by controlling the shape and surface pattern of the hydrogels and modulating the position, time, and motion of the laser of NIR light. For example, a hand-shaped hydrogel matrix was designed, and then the NIR light was sequentially illuminated to bend each fingers at the desired location (Fig. 7b). They also synthesized a hydrogel actuator on a bent substrate to generate an arch-shaped gel, and the NIR was exposed to the edge of the hydrogel. This causes the front edge to rise up and the back edge to push the glass as the hydrogel curled, resulting in forward crawling. The gel continued at approximately 3 mm per cycle. Unlike previous hydrogel actuators, the ELP–rGOs hydrogels did not require additional chemical reagents or ratchet substrates [69].

rGOs can be utilized for improvement of mechanical property, and also rapid electro-responsiveness of the hydrogels. Yang et al. [58] proposed poly(2-acrylamido-2-methyl-propanesulfonic acid) hydrogels comprising rGOs (poly(AMPS-*co*-AAm)/rGOs) (Fig. 7c). The rGOs uniformly dispersed in the hydrogel can provide an excellent conductive platform to promote ion transport within the hydrogel, thereby generating a significant osmotic pressure between the exterior and interior of the nanocomposite hydrogel. Therefore, the speed of electrical response and volume change of the hydrogels become rapid and remarkable. Additionally, the mechanical properties, including tensile strength and compressive strength, of the poly(AMPS-*co*-AAm)/rGOs hydrogels are enhanced by the hydrogen bonding interaction between the rGOs and polymer chains. Figure 7d shows that the poly(AMPS-*co*-AAm)/rGOs hydrogels exhibit reversible bending behaviors in electrolyte solutions. When applying the electric field for 2 min, the hydrogels shrank to 58–68% of their original weight. After removing the electric field, they recovered their initial state within 6 min. These results indicate the rapid and reversible electro-induced swelling–deswelling properties of these nanocomposite hydrogels. Moreover, the responsive rate and degree can be controlled by the contents of the rGOs.

The anisotropic internal structures are well known to induce large and directional deformation. Hence, Xu et al. [59] developed a new type of PNIPAAm/GOs hydrogel by applying DC electric field to induce gradually oriented GOs into the thermoresponsive hydrogel. A one-pot synthesis strategy was performed to achieve the desired form of the PNIPAAm/GOs hydrogel, as shown in Fig. 7e. When DC field was applied, negatively charged GOs moved to the anode site during electrophoresis. Simultaneously, PNIPAAm chains were chemically cross-linked by *N,N*′-methylene bis(acrylamide) (BIS), whereas oxygen-containing GOs and amide groups of PNIPAAm chains were physically cross-linked by hydrogen bonding. Consequently, in the hydrogel, the gradually oriented GOs were obtained along the direction from the cathode to anode side based on the DC direction. Figure 7f illustrates the hydrogel lifted loading cargo successfully after 30 s of NIR irradiation (808 nm).

#### TiNSs

**Fig. 8:**
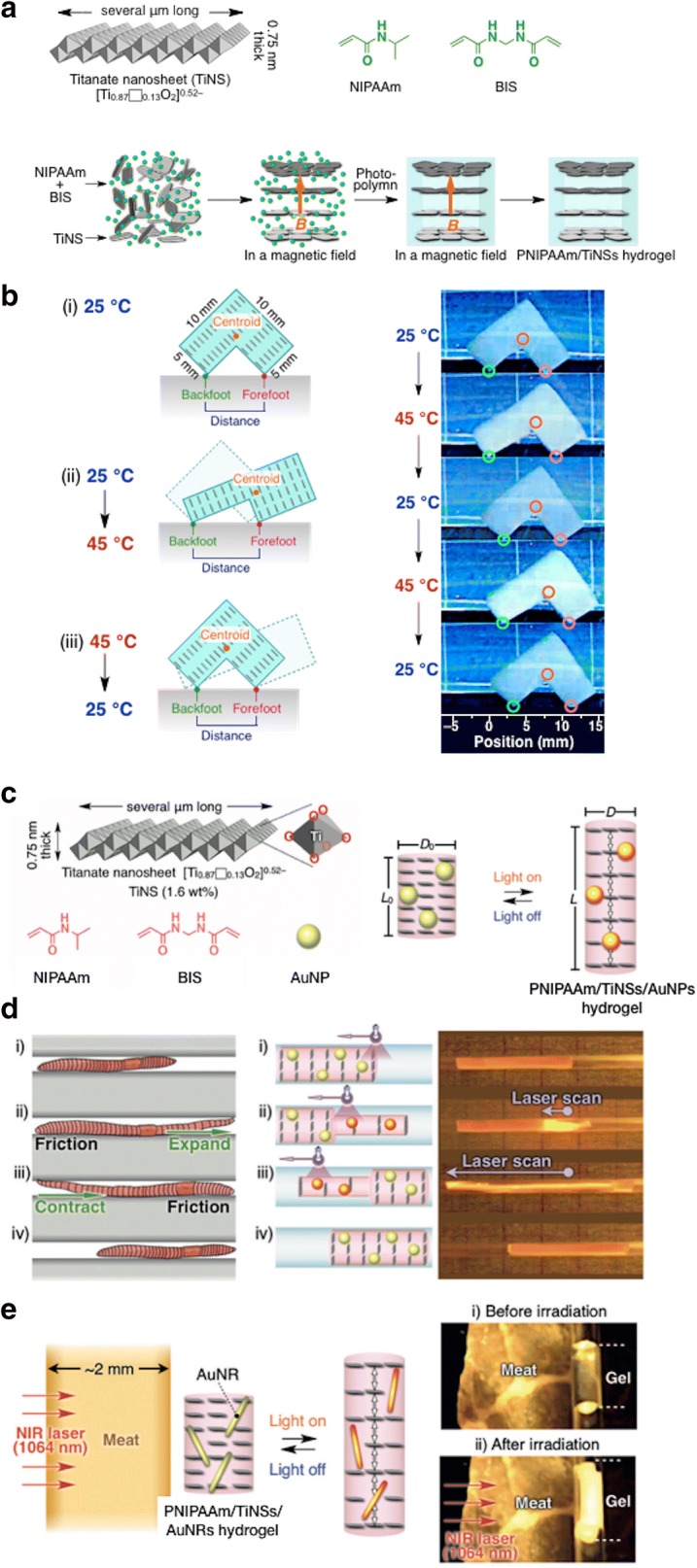
**a** Scheme of preparation of the PNIPAAm/TiNSs hydrogel with cofacially oriented TiNSs in a magnetic flux of 10 T using a the UV curing method. **b** Demonstration of the thermoresponsive deformations with unidirectional procession of an l-shaped symmetric PNIPAAm/TiNSs hydrogel actuator. (**a**, **b** were reproduced with permission from Ref. [60], © 2015 Nature Publishing Group). **c** Synthesis components of the photo-responsive PNIPAAm/TiNSs/AuNPs hydrogel actuator. Magnetic flux of 10 T was used for cofacially oriented TiNSs/AuNPs in a cylindrically processed hydrogel. **d** Schematic of the peristaltic craving of an earthworm-like hydrogel. These optical images were captured while the laser was scanned with 445 nm laser light (5.6 W/cm^2^ power density). **e** Hydrogel fabrication by using TiNSs/AuNRs, instead of AuNPs. The images show the hydrogel that is responsive to NIR light irradiated through a ≈ 2-nm-thick piece of meat. The optical images also illustrate the pre- and post-irradiation with 1064 nm NIR laser light (12 W/cm^2^ power density). (**c**–**e** were reproduced with permission from Ref. [72], © 2018 Wiley–VCH Verlag GmbH & Co. KGaA, Weinheim)

TiNSs can provide considerable and unique properties dominated by the anisotropic–electrostatic repulsion between themselves [70]. Kim et al. [60] applied cofacially aligned TiNSs into the PNIPAAm hydrogel. They reported that when the gel network was constructed by PNIPAAm, the LCST behavior reversibly modulated the anisotropic–electrostatic repulsion between cofacially aligned TiNSs in the hydrogel matrix [71]. To prepare the PNIPAAm/TiNSs hydrogel, the aqueous dispersion of TiNSs was firstly placed in a magnetic field (10T), and then the in situ radical polymerization was conducted to fix the anisotropic orientation of TiNSs. TiNSs were arranged cofacially to one another to ensure the maximal electrostatic repulsion between them (Fig. 8a). The PNIPAAm/TiNSs hydrogel exhibited opposite thermal response behavior, compared to the conventional PNIPAAm hydrogel. Thermal motion of their PNIPAAm/TiNSs hydrogel occurred without substantial water uptake and release. Therefore, unlike the conventional PNIPAAm hydrogels, actuation of PNIPAAm/TiNSs hydrogel could be performed in open-air system, and also in non-aqueous media. The deformation rate observed in this research (∼ 70% s^−1^) was the largest record among those reported for stimuli-responsive hydrogels. Moreover, they attempted to use a hydrogel actuator as an L-shaped symmetrical bipedal walking object, designed to allow the forefoot and backfoot to be in contact with the horizontal, flat base. l-Shaped actuator proceeded unidirectionally upon repeatable heating and cooling (Fig. 8b).

Additionally, Sun et al. [72] reported an anisotropic hydrogel actuator that generated earthworm-like peristaltic crawling. Figure 8c illustrates the synthesized hydrogel with cylindrical shape containing AuNPs as the photo-thermal convertors, thermoresponsive polymer network (PNIPAAm) for permittivity switching of the gel interior, and cofacially oriented 2D material TiNSs to adjust their anisotropic–electrostatic repulsion. The researchers monitored the temperature change of the PNIPAAm/TiNSs/AuNPs hydrogel using a thermal camera during irradiation of 445 nm laser light (power density of 5.6 W/cm^2^). The result showed that the irradiation for only 30 s could increase the hydrogel temperature up to 85 °C. Meanwhile, the irradiation of the NIR light lower than 1 s was sufficient to improve the temperature above the LCST. This photo-thermal conversion was also repeatable without any loss of its efficiency by turning on and off the laser light. In contrast, the reference hydrogel without AuNPs did not exhibit any critical temperature change. Within this tremendous change material, they demonstrated that the PNIPAAm/TiNSs/AuNPs hydrogels performed earthworm-like peristaltic crawling. As the NIR-irradiated region of the hydrogels spatiotemporally became long and thin, the friction on the capillary wall was thereby reduced (Fig. 8d). For the crawling movement, the irradiation spot was moved toward the left end with a velocity of 3.4 mm/s. Additionally, the hydrogel actuator containing TiNSs/Au nanorods (AuNRs) was synthesized, where AuNRs can convert the 1064 nm laser light into thermal energy. They demonstrated that penetrated NIR laser light through a ~ 2-mm-thick piece of meat could cause the deformation of the PNIPAAm/TiNSs/AuNRs hydrogel within 6 s (Fig. 8e). Therefore, by means of the anisotropic structure, a rapid, large, repeatable, spatiotemporal, and anisotropic photo-thermal deformation of the hydrogel was possible.

#### TMDs

Recently, TMDs have elicited increasing interest because of their tremendous properties, such as high surface area, large band gap, and unique optical characteristics. Thus, TMDs have a remarkable potential in biological applications, electrocatalysis, and optoelectronic devices. Although they have been utilized in outstanding studies, specific cooperation with functional polymers is still required.

**Fig. 9:**
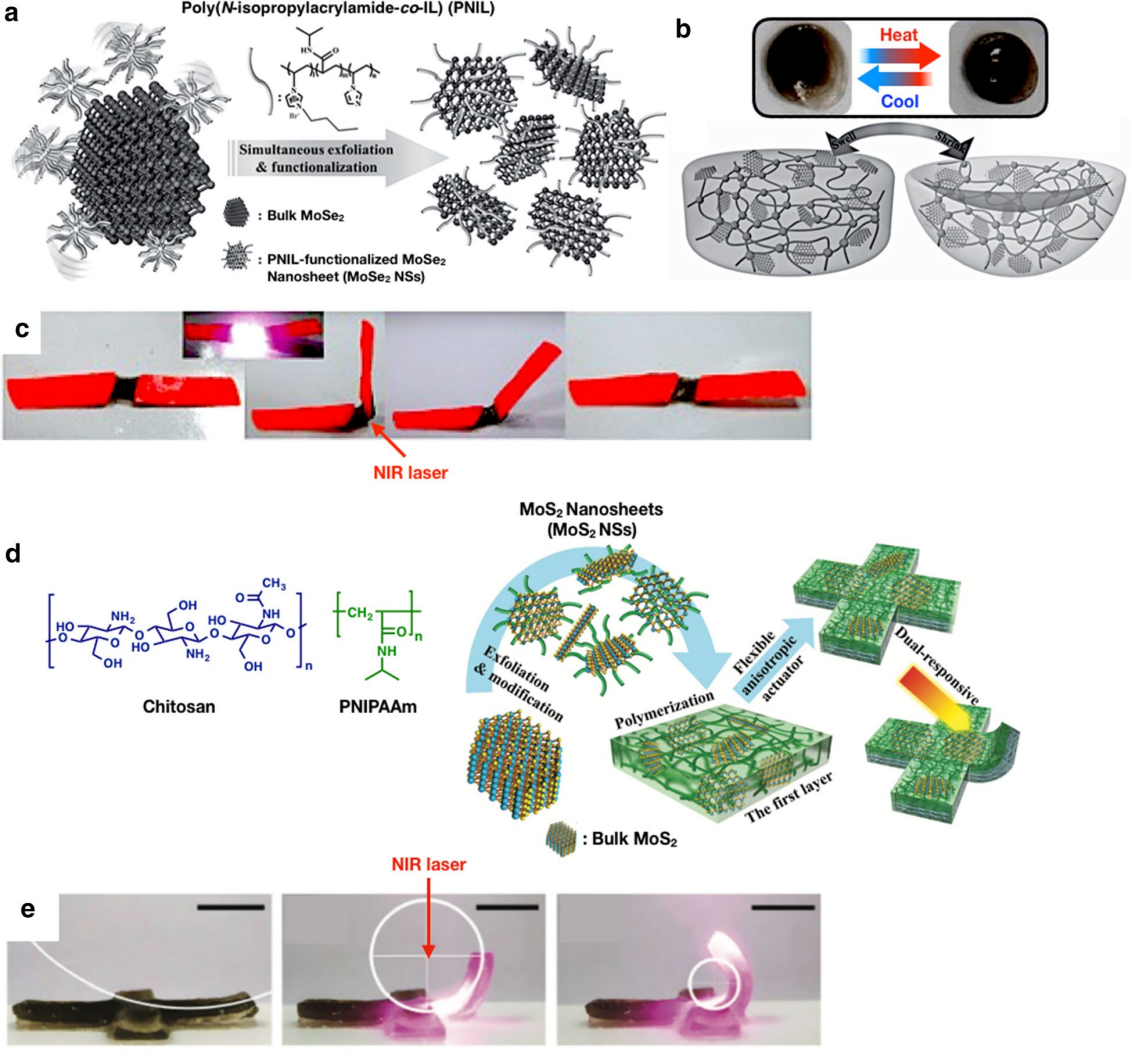
**a** Overall preparation scheme of the simultaneous exfoliation and noncovalent functionalization of MoSe_2_ NSs with PNIL. **b** Image of the MoSe_2_ NSs immobilized hydrogel at 25 °C and 55 °C. **c** Image of the bending of the MoSe_2_ NSs immobilized hydrogel upon 808 nm irradiation for 3 min. (**a**–**c** were reproduced with permission from Ref. [61], © 2016, Wiley–VCH Verlag GmbH & Co. KGaA, Weinheim). **d** Schematic images of the exfoliation and modification of MoS_2_ NSs with chitosan and synthesis of the bilayered hydrogel composed of PNIPAAm/MoS_2_ NSs layer and chitosan/DMA/LAPONITE layer. **e** Images of the response of the MoS_2_ NSs immobilized hydrogel under 808 nm NIR irradiation (5 W/cm^2^) for 70 s. (**d**, **e** were reproduced with permission from Ref. [62], © 2016, The Royal Society of Chemistry)

Lei et al. [61] first introduced TMDs in 2016 to multifunctional hydrogels. Previously, only few exfoliation studies on the TMDs functionalization had been reported [73]. However, the previously reported methods have the following limitations: availability of specific ligands, complex reactions, and harsh environments. Therefore, they developed appropriate functional polymers to facilitate the TMDs exfoliation. Then, the resultant functionalized TMDs can be directly integrated into several novel nanocomposites with excellent multifunctional applications. In this work, they attempted to cooperate a polymeric ionic liquid (PIL) with thermosensitive monomer (poly(*N*-isopropylacrylamide-*co*-IL), PNIL) to functionalize molybdenium diselenide nanosheets (MoSe_2_ NSs) (Fig. 9a). Considering that imidazole-based PIL interact effectively with the MoSe_2_ NSs, this PIL has been selected for an effective dispersion agent of MoSe_2_ NSs. Through a one-step cross-linking reaction by PNIL, the resultant PNIL-functionalized MoSe_2_ NSs were assembled into dual photo- and thermoresponsive hydrogels. Moreover, MoSe_2_ NS was regarded as a practical photo-thermal agent with high optical absorbance in the NIR region. Consequently, the temperature increased from 5.9 to 28.1 °C under 808 nm laser irradiation. This indicates that the photo-thermal conversion efficiency of the PNIL-functionalized MoSe_2_ NSs was calculated to be 54.1%, among the highest records on the reported NIR photo-thermal nanotransducers. As shown in Fig. 9b and c, the stimuli-response of the hydrogel was reversible and could be remotely controlled. When the laser was turned on (808 nm, 2.5 W/cm^2^), the temperature of the MoSe_2_ NSs rose and then the nanocomposite hydrogel bent. When the laser irradiation was turned off, the hydrogel stretched back to its initial state.

In the same research group, Lei et al. [62] reported flexible anisotropic actuators that are Molybdenium disulfide nanosheets (MoS_2_ NSs) based and dual responsive. MoS_2_ NSs acted as the photo-thermal transduction agents during the NIR irradiation (808 nm), the same as the previously described MoSe_2_ NSs. A smart flexible actuator was developed by utilizing biomacromolecule-functionalized MoS_2_ NSs, tough hydrogel matrix with tunable volume phase transition temperature (VPTT), and well-designed anisotropic architecture. Chitosan was used for the exfoliation of the MoS_2_ NSs, rendering MoS_2_ NSs to be hydrophilic. Thus, the as-prepared nanosheets became remarkably stable in water (Fig. 9d). Owing to the good dispersibility of MoS_2_ NSSs in water, inorganic–organic hybrid hydrogels could be easily obtained, resulting in the poly(*N*-isopropylacrylamide)-*co*-dimethylacrylamide (PNIPAAm-*co*-DMA) composite hydrogel. The PNIPAAm-*co*-DMA/MoS_2_ NSs hydrogel was synthesized with bilayer system. First layer was composed of PNIPAAM and MoS_2_, and the second layer was formed by chitosan, DMA and LAPONITE. After the NIR light was irradiated, the hydrogel temperature reached the VPTT in the first layer at 10 s, causing the bending of the entire hydrogel (Fig. 9e). Therefore, the bilayered structure rendered the hydrogels produce a smart behavior in shape deformation and self-wrapping with remotely controlled light or heat.

#### FHT LC NSs

**Fig. 10:**
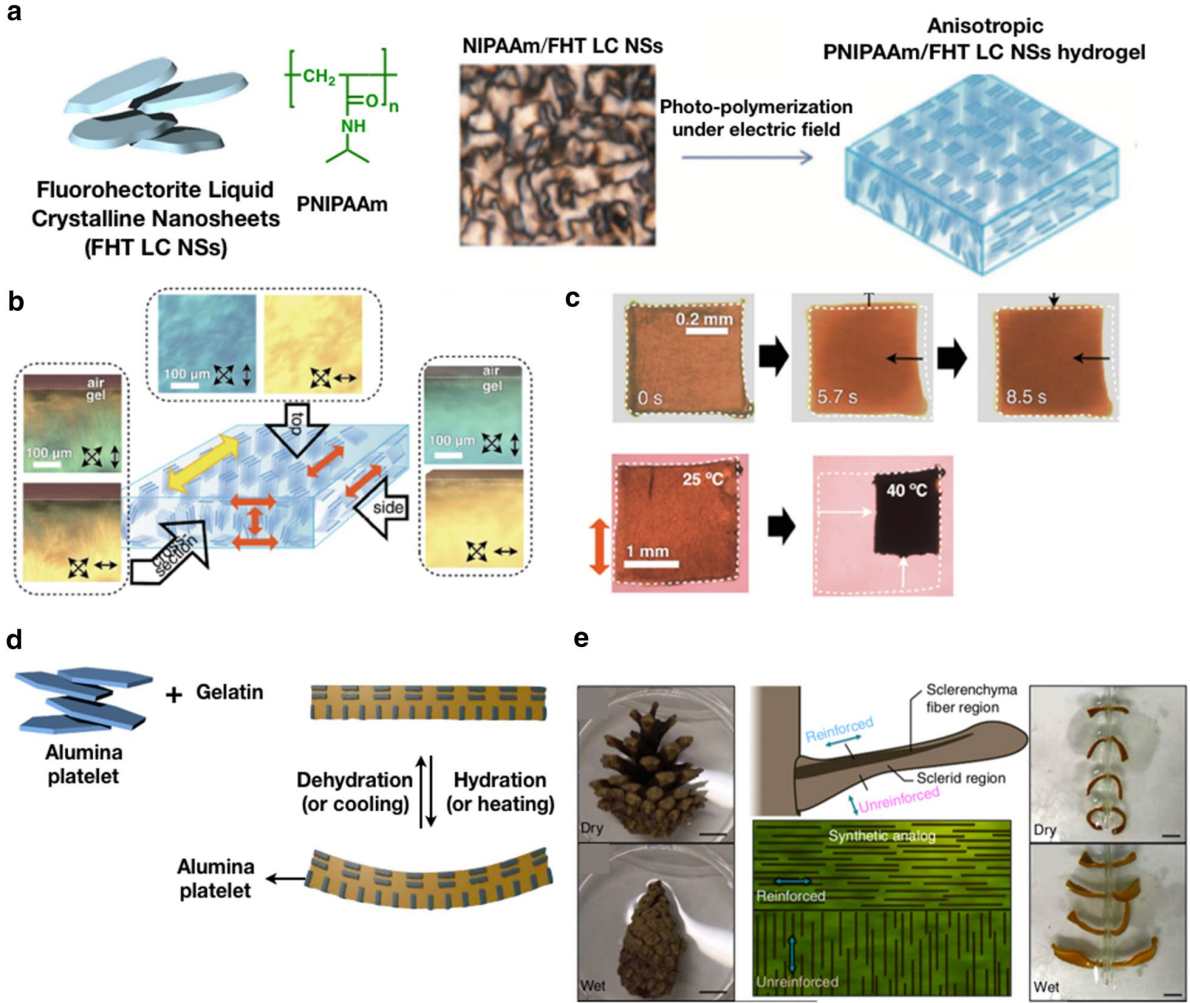
**a** Schematic images of preparation of the anisotropy hydrogel with FHT LC NSs and PNIPAAm under electric field. **b** Microscopic view of the PNIPAAm/FHT LC NSs hydrogel synthesized with in-plane and out-of-plane electric field. The optically polarized images are observed with crossed polarizers and a wave plate. **c** Photographs of the anisotropic deformation of the PNIPAAm/FHT LC NSs hydrogel film upon photo-irradiation (top) and heating (bottom). (**a**–**c** were reproduced with permission from Ref. [63], © 2014, Wiley–VCH Verlag GmbH & Co. KGaA, Weinheim). **d** Schematic of the hydrogel actuator comprising structurally oriented alumina platelets and gelatin. **e** Schematic images of the behavior of pinecone in dehydration/hydration process and the predominant orientation of CMFs within a pinecone scale (left). Synthetic pinecone scales constructed by oriented platelets in a bilayer structure within gelatin (right). (**d** was reproduced with permission from Ref. [65], © 2013 Macmillan Publishers Limited)

FHT LC NSs are novel inorganic materials with excellent electrical, magnetic, and mechanical properties. Recently, Inadomi et al. [63] succeeded in constructing a thermoresponsive PNIPAAm hydrogel doped with monoaxially aligned FHT LC NSs and photo-active dyes (Fig. 10a). From the images of the polarized optical microscopy, a interference color of blue or yellow was observed in the top and side images of the aligned nanocomposite hydrogel. This indicates that the FHT LC NSs were aligned along the applied electric field (Fig. 10b). Consequently, unlike conventional PNIPAAm hydrogels that exhibit isotropic swelling and deswelling upon cooling and heating, respectively, the proposed FHT LC NSs composite hydrogel demonstrated anisotropic shrinkage and anomalous expansion in a specific direction upon exposure to light (Fig. 10c).

#### Alumina platelets

Erb et al. [65] proposed a hydrogel actuator with programmable bio-inspired microstructures. The presented complex hydrogel actuators were inspired by the bending and twisting mechanisms represented by pinecones. In these particular structures, bending and twisting can be performed, assisted by the structurally oriented rigid cellulose microfibers (CMFs). To mimic this structure, gelatin-based hydrogels comprising magnetically oriented alumina platelets were synthesized (Fig. 10d). The alumina platelets can be oriented through magnetic fields because they had been coated with superparamagnetic IONPs. Such platelets have a distinct geometry relative to CMFs, offering 2D reinforcement. The researchers demonstrated an artificially replicated bilayer structure exhibited by pinecone that bent during dehydration/hydration (Fig. 10e). The orientations of platelets in the upper and lower layer of bilayered hydrogel were different from each other, so their deformation exhibited in different ways with hydration or dehydration. By systematically adjusting the platelets orientation angles in the upper and lower layers, deformation modes such as curling and clock- and anticlockwise twisting were programmed.

Table 1 summarizes the maximum deformability, response speed, and response efficiency of the hydrogel actuators, according to the implanted additive materials, composing polymers, and applied stimuli.

**Table 1:** Classification of additives, polymers, and required stimuli of the hydrogel actuators and actuation comparisons in terms of their maximum deformability, response speed, and response efficiency

Dimensions	Material	Polymer	Stimuli source	Release energy	Maximum deformability	Response speed	Response efficiency	Refs.
Zero dimension (0D)	Gold nanoparticle and nanoshell (AuNP and AuNS)	Poly(*N*-isopropylacrylamide) (PNIPAAm)	Near Infrared light (NIR)	Heat	4.28 cm^−1^ [46]	5 s [35], 25 s [42], 20 min [46]	–	[35, 42, 46]
	Iron oxide nanoparticle (IONP)	PNIPAAm	Alternating magnetic field (AMF)	Heat	–	–	–	[46]
		Acrylamide (AAm)	Magnet		0.9 cm^−1^	–	–	[37]
	Ytterbium and neodium	PNIPAAm	808 and 980 nm light irradiation	Heat	45° bending	< 10 s	–	[39]
	Poly(dopamine) nanoparticle	PNIPAAm	MR light	Heat	–	30 s	77%	[40]
One dimension (ID)	Nanofibrillated cellulose	*N*,*N*-dimethylacrylamide	Hydration/dehydration					[50]
	Thermoplastic urethane (TPU)	PNIPAAm	Temperature change		–	0.6 s	–	[51]
	Carbon nanotube (CNT)	PNIPAAm	Temperature change		90° bending	~ 2.7 s	–	[52]
	Multiwall carbon nanotube (MWCNT)	Polyvinylalcohol (PVA)	Electric field	Heat	58° bending	30 s	–	[53]
Two dimension (2D)	Reduced graphene oxide (rGO)	Elastin-like polypeptide (ELP)	NIR light		60° bending	3 s	–	[57]
		Poly(2-acrylamido-2-methyl-propanesulfonic	Electric field	Heat	0.088 mm^−1^	12 s	–	[58]
	Graphene oxide (GO)	PNIPAAm	NIR light		300° bending	40 s	–	[59]
	Titanate nanosheet (TINS)	PNIPAAm	Temperature change	Heat	170% elongation	In a second	~ 70% s^−1^	[60]
	TiNS + AuNP	PNIPAAm	NIR light	Heat	180% elongation	< 0.5 s	–	[72]
	Transition metal dicalchogenide (TMD)	PNIPAAm	NIR light		0.22 mm^−1^ [62]	70 s [62]	–	[61, 62]
	Fluorohectorite (FHT)	PNIPAAm	Temperature change		130%	5.6 s	–	[63]

## Conclusion

In this review paper, we have briefly introduced various nanocomposite hydrogel actuators sorted by dimensions of additive functional organic/inorganic materials. Each of these nanocomposite hydrogel actuators induces distinct behaviors based on several stimuli, such as pH, heat, light, electric field, and magnetic field.

By incorporating several organic or inorganic additives to hydrogels, the limitations of stimuli-responsive hydrogel actuators, such as slow response, small deformation, and low mechanical property, were generally addressed. Multiple hybridization of different materials including 0D-, 1D-, 2D additives and functional polymers would give rise to synergic effects, allowing much improved performance of actuators and also generation of new functions.

Additive materials are not only useful in various stimuli-responsive mediators but also aid in mechanical property improvement of hydrogel actuators. For the development of flexible and mechanically durable hydrogel actuators, physical or mathematical modeling of the polymers and additives, in terms of complementarity, should be implemented. Furthermore, the 3D printing technique could offer a new avenue to make the innovative 3D hydrogel actuators.

In order to realize hydrogel actuators to be applied to future artificial muscles or soft robots, new functions of nanocomposite materials must be derived and manufacturing techniques also should be further developed. We hope this review article can be used as guide in selecting nanocomposites for synthesizing novel hydrogel actuators.

## Data Availability

The review is based on the published data and sources of data upon which conclusions have been drawn can be found in the reference list.
